# Factors associated with awareness, reading and perceptions of clinical practice guidelines among the general population in Japan: A nationwide cross-sectional survey (INFORM Study 2023)

**DOI:** 10.1371/journal.pone.0343033

**Published:** 2026-02-12

**Authors:** Yosuke Hatakeyama, Akiko Yaguchi-Saito, Kanako Seto, Ryo Onishi, Ryosuke Hayashi, Koki Hirata, Kunichika Matsumoto, Tomonori Hasegawa, Yoshimitsu Takahashi, Takeo Nakayama, Junko Saito, Aki Otsuki, Aya Kuchiba, Naomi Sakurai, Naoki Nakaya, Maiko Fujimori, Taichi Shimazu, on behalf of the INFORM Study 2023 Group

**Affiliations:** 1 Department of Social Medicine, Toho University School of Medicine, Tokyo, Japan; 2 Faculty of Human Sciences, Tokiwa University, Ibaraki, Japan; 3 Division of Behavioral Sciences, National Cancer Center Institute for Cancer Control, National Cancer Center, Tokyo, Japan; 4 Department of Implementation Science in Public Health, Kyoto University School of Public Health, Kyoto, Japan; 5 Department of Health Informatics, Kyoto University School of Public Health, Kyoto, Japan; 6 Graduate School of Public Health, Teikyo University, Tokyo, Japan; 7 Division of Biostatistical Research, Institution for Cancer Control/Biostatistics Division, Center for Research Administration and Support, National Cancer Center Hospital, Tokyo, Japan; 8 Cancer Solutions, Inc, Tokyo, Japan; 9 Tohoku Medical Megabank Organization, Tohoku University, Miyagi, Japan; 10 Division of Survivorship Research, National Cancer Center Institute for Cancer Control, National Cancer Center, Tokyo, Japan; King Saud University Medical City, SAUDI ARABIA

## Abstract

**Background:**

Clinical Practice Guidelines (CPGs) support evidence-based, shared decision-making by providing recommendations based on the best available evidence, including systematic reviews, clinical trials, and expert consensus. Despite their potential to improve the quality of healthcare and reduce unnecessary interventions, CPGs remain inadequately disseminated and implemented. Key barriers include limited awareness and poor perception among patients and the general public. To inform strategies to improve CPG dissemination, this study aimed to investigate the association between socioeconomic factors and the awareness, reading, and perceptions of CPGs among the Japanese general population.

**Methods:**

A nationwide cross-sectional anonymous mail survey (INFORM Study 2023) was conducted among 10,000 randomly selected Japanese adults aged ≥20 years using stratified two-stage random sampling. This study was conducted as part of the INFORM Study 2023, with items related to CPGs included as an approved module. Logistic regression models were used to identify respondent characteristics associated with the awareness and reading experience of CPGs, and to assess the association between reading CPGs and eight perceptions held about them.

**Results:**

The response rate was 35.3% (n = 3,452). After excluding cases with missing data on CPG awareness, the remaining responses were included in the analysis (n = 3,343). Among these, 82.5% were unaware of CPGs, 9.9% were aware of CPGs but had never read one, and 7.6% were aware of CPGs and had read one. Awareness was significantly higher among individuals with higher education (adjusted odds ratio [aOR]: 2.060 for vocational school; 1.813 for university graduates), higher household income (aOR: 1.882 for ≥8 million yen), excellent health (aOR: 1.373), and frequent healthcare visits (aOR: 1.741 for ≥10 visits/year). Household size of ≥2 members (aOR: 0.591) was associated with lower awareness. Reading CPGs was associated with a self-history of cancer (aOR: 1.858). Of eight perceptions, respondents who had read CPGs correctly disagreed with the notion that CPGs harm patient-provider relationships. Among other respondents, misconceptions about CPGs persisted, particularly regarding their limitations and recommendations.

**Conclusions:**

Among the general population in Japan, awareness of CPGs is associated with demographic and socioeconomic characteristics. Tailored dissemination strategies—particularly for individuals with lower income or education levels and other groups identified in this study—are essential to improving awareness and supporting informed healthcare decision-making. These findings also highlight the need to integrate CPG literacy into national health education initiatives to enhance public access to evidence-based care.

## Background

Clinical practice guidelines (CPGs) are documents which aim to support shared decision-making by providing recommendations based on the systematic review of existing evidence and assessment of the benefits and harms of alternative care options [1]. In Japan, CPGs have been developed since around 2000 and disseminated in books, papers, and on websites. Further, CPGs for patients and the general public have also been published. Among their potential benefits, CPGs can support clinician–patient decision-making, improve the quality of care, and inform the development of patient education materials. They also improve patient outcomes and reduce unnecessary interventions. [2–5]. To achieve their benefits, CPGs must be effectively disseminated and implemented; however, previous studies have shown that these processes remain insufficient [6–11]. Barriers to dissemination and implementation include factors attributable to medical professionals and to CPGs themselves, as well as patient factors, including knowledge of their condition and its management, health literacy, and awareness of CPGs [12–14]. Previous studies have shown that awareness of CPGs is associated with a more accurate understanding of their purpose, recommendations, and limitations, whereas low awareness is linked to persistent misconceptions among the public and patients [15–17].

Studies on the public awareness, reading, and perceptions of CPGs reported to date have primarily focused on patients at medical institutions and individuals actively seeking medical information [15]. A survey of public awareness and use of CPGs in Japan also focused on people who had received medical care within the past three months [18]. Although these studies did not examine the general population, CPGs are applied not only in clinical practice but also as a basis for understanding health policy decisions, legal proceedings, and media discussions on optimal healthcare practices [1,19–21]. Therefore, enhancing public awareness of CPGs could empower individuals to critically evaluate CPG-related information and make informed decisions when facing medical situations as patients. However, little attention has been paid to the awareness of CPGs in the general population.

Despite the growing emphasis on shared decision-making and evidence-based care, little is known about how the general public in Japan understands or engages with CPGs. Existing studies have primarily focused on patients receiving medical care or individuals who actively seek health information, leaving substantial gaps in knowledge about CPG awareness among the broader population. A detailed understanding of public awareness and perceptions is essential, as effective dissemination and correct comprehension of CPGs may support informed decision-making, reduce misunderstanding, and ultimately promote equitable use of evidence-based healthcare.

Given these gaps, this study aimed to examine factors associated with awareness, reading, and perceptions of CPGs among the general population in Japan. Specifically, we addressed two research questions: (1) Which demographic and socioeconomic groups remain unaware of CPGs or have never read them, given that identifying these groups is necessary for designing targeted dissemination strategies; and (2) which aspects of CPGs are difficult for the public to correctly understand, even among those who have read them, given that clarifying these areas can guide the development of effective educational materials and communication strategies. By answering these questions, this study aimed to provide foundational evidence for improving national-level dissemination and understanding of CPGs.

## Materials and methods

In May 2023, we conducted a nationally representative cross-sectional survey on health information access for consumers in Japan (INFORM Study 2023) [22], focusing on “Living with Cancer.” The mailing envelope contained a document describing the study’s aims, and a questionnaire which included a statement requesting consent to participate in the study. Participation was voluntary, and informed consent was obtained by asking respondents to checkmark a consent statement on the questionnaire and return it anonymously by mail. We sent a reminder to nonrespondents by postcard and sent a 500 yen (US$3.33) cash voucher to all respondents.

The present study was conducted as part of the INFORM Study 2023. This is one wave of the ongoing INFORM Study project that investigates how the Japanese population accesses and uses health information. INFORM Study 2020 focused on cancer prevention and screening [23–30] and INFORM Study 2023 focused on living with cancer. The two studies were carried out in different years. They included both common modules (health information seeking, trust in information sources, communication with healthcare providers, general health status, and cancer history) and wave-specific questionnaire modules (awareness and perceptions of CPGs, balancing cancer treatment and work, hereditary cancers, fertility and reproductive concerns, and awareness, information sources, and beliefs regarding palliative care [22]). They are successive waves of the INFORM Study Project and therefore share an identical sampling framework, stratification scheme, and data collection procedures. For this reason, methodological details of the sampling design are referenced from the INFORM Study 2020 protocol [23], which provides the most comprehensive description of the common survey framework. The INFORM Study 2023 questionnaire received separate ethical approval from the Research Ethics Committee of the National Cancer Center (Approval No. 2022–360). The present analyses are based solely on the INFORM Study 2023 dataset.

### Sample size

Sample size calculations in this study were performed using the recommended margin of error (0.05) from the World Health Organization Sample Size Calculator (https://cdn.who.int/media/docs/default-source/ncds/ncd-surveillance/steps/sample-size-calculator.xls) and the same parameter settings as in the INFORM Study 2020 [23], assuming a response rate of 35% based on the previous actual response rate (37.1%) [24–29]. When respondents were divided into quintiles, the sample size for each group was calculated as 2195.2, as follows:

N = 1.96^2^ × 0.5 × (1–0.5)/ 0.05^2^ × 2/ 0.35 = 2195.2,

where 1.96 is the level of confidence measure, 0.05 is the margin of error, 0.5 is the baseline level of the indicators, 2 is the design effect, and 0.35 is the expected response rate.

Accordingly, the sample size required for the five groups was 10,976 (n = 2195.2 × 5 = 10,976), and the final sample size was set at 10,000.

### Target population

The target population was members of the Japanese general population aged ≥20 years who were registered in basic municipal resident register. The sampling strategy was the same as that used in the INFORM Study 2020 [23]. Individuals from the general Japanese population were sampled using two-stage stratified random sampling. Sampling strata were defined by crossing nine geographic regions of Japan with four categories of municipality size based on population (city size), yielding a total of 35 strata. Municipality size was used to account for urban–rural differences. In the first stage, 500 census enumeration areas were selected as primary sampling units with probabilities proportional to population size across strata. In the second stage, individuals aged 20 years or older were sampled within each selected census enumeration area, using the Basic Resident Register as the sampling frame. A stratified two-stage random sampling design was used to reduce the risk of chance underrepresentation of smaller-population areas and thereby reflect the distribution of regions and municipality sizes in the target population, and to improve sampling efficiency.

### Survey measurement

Questionnaire items were mainly selected from the Health Information National Trends Survey (HINTS) conducted in the United States to ensure comparability [22,31]. The common components of the questionnaire used in the INFORM Study Project were based on the original HINTS items [23], which were translated into Japanese and then back-translated into English. The back-translation was reviewed and confirmed by the HINTS developers to ensure conceptual and semantic equivalence. Additional items related to CPGs were newly developed for the INFORM Study 2023. To ensure content validity, the CPG items underwent internal expert review by specialists in guideline development, health communication, and survey methodology within the INFORM Study 2023 team. Cognitive interviewing was conducted with 16 members of the general public to assess clarity, comprehension, and response processes. Participants were asked whether the intent and wording of each item were easy to understand, and items that were unclear were revised accordingly.

The items related to CPGs consisted of three domains: (1) awareness of CPGs (1 item), (2) reading of CPGs (1 item), and (3) perceptions of CPGs based on previous studies that cited common public misperception about CPGs [15,16,32] (8 items). A detailed list of the CPG-related items is provided in Supplementary Material (S1 File).

These eight items for perception of CPGs had five possible responses: (i) Strongly agree, (ii) Somewhat agree, (iii) Don’t agree so much, (iv) Don’t agree at all, and (v) Don’t know (DK). Of these responses, (iii) and (iv) were recorded into “Disagree”, which meant that the respondent had correctly denied the misconception. The other responses – (i), (ii), and (v) – were grouped into the same category as “Agree/DK”. Respondents’ inability to accurately reject incorrect statements was interpreted as a potential indicator of limited or uncertain conceptual understanding of CPGs. This approach is consistent with established methods in health literacy research, in which difficulty identifying incorrect statements is used as a marker of incomplete understanding rather than merely a wrong answer [33].

The following participant characteristics were collected: age; gender; education; marital status; number of household members; employment status; annual household income; general health; number of healthcare visits per year; history of cancer, either themselves or their family or friends; and experience with a diagnosis of diabetes, hypertension, heart disease, cerebrovascular disease, chronic lung disease, arthritis, and depression. These were demographic characteristics investigated in a previous study on health literacy [26] and illness experiences assessed in a study investigating awareness of CPGs in Japan [18]. Experience of diseases was asked about in a yes/no format and responses were not mutually exclusive.

### Analysis

To account for the complex sampling design and non-responses among the Japanese general population, weighted analysis was performed to accurately calculate population parameter estimates. We calculated the weight for each respondent by multiplying the sampling weights and non-response weight. Detailed information is described in a previous paper [23].

We divided respondents into three groups: 1) those who were not aware of CPGs, 2) those who were aware of CPGs but had never read one, and 3) those who were aware of CPGs and had read one. Subsequently, we assessed the relationship between respondents’ characteristics and their familiarity with CPGs using cross-tabulation and chi-square tests.

A weighted, multivariable logistic regression model was used to evaluate the associations between respondent characteristics and awareness of CPGs, as well as between them after excluding healthcare-related variables that were not significant in a previous analysis and experience of reading CPGs. Associations between the experience of reading CPGs and perceptions of them were assessed by a logistic regression model with adjustment for respondent characteristics. Statistical significance was set at 0.05, and all tests were two-tailed. IBM SPSS Statistics (version 29) was used for all analyses.

## Results

### Characteristics of respondents

**Fig 1:**
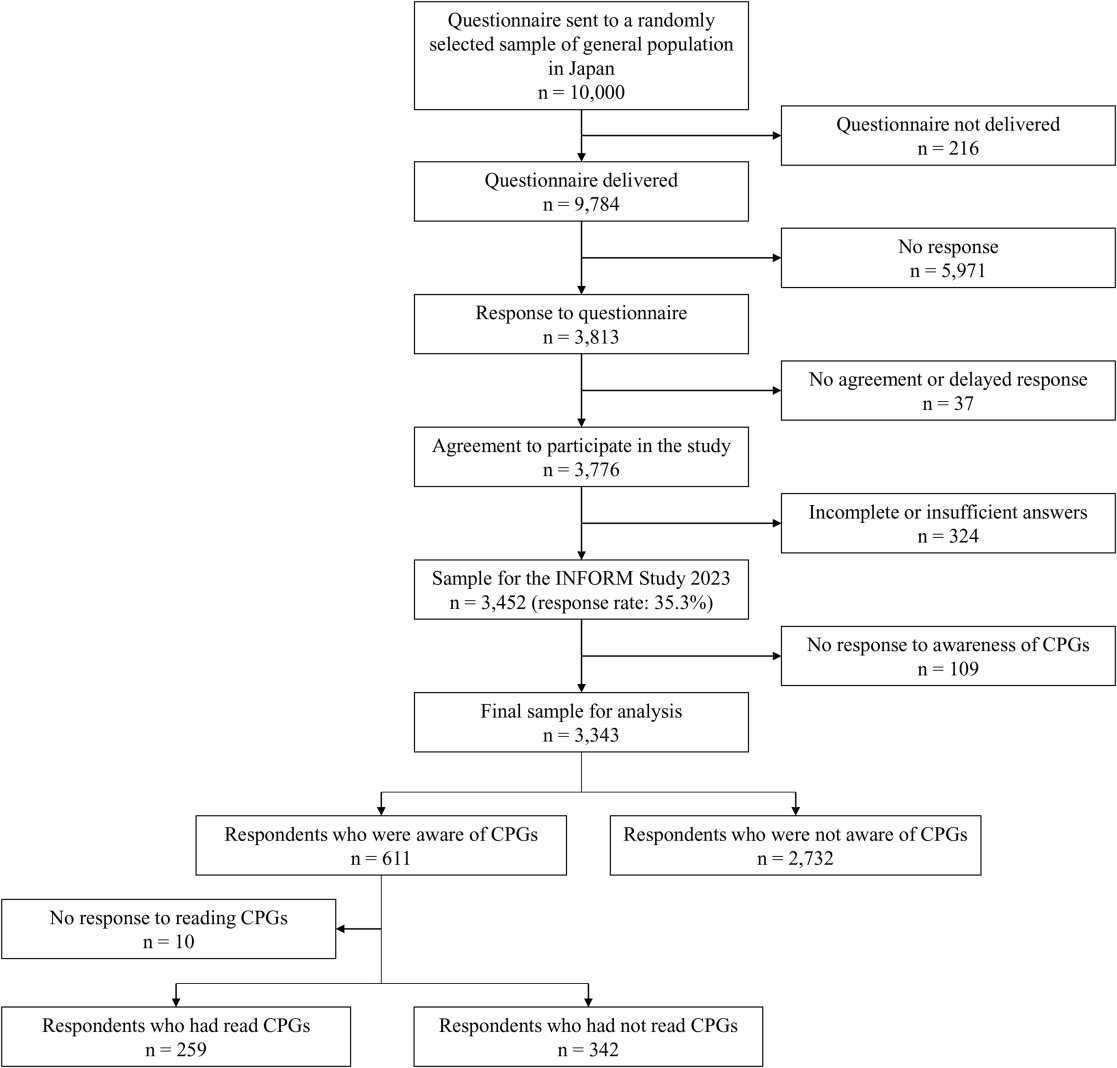
Flowchart of study participants. CPG; clinical practice guideline.

The questionnaires were sent to randomly sampled people in the general population in Japan (n = 10,000). After exclusion of 216 cases due to non-delivery of the questionnaire, the target population size was 9,784. In total, 3,776 people responded to the survey and agreed to participate in the survey. After dealing with missing values and data cleaning, 3,452 participants were included in the INFORM Study 2023, giving a response rate of 35.3% (3,452/9,784). Subsequently, responses with missing values concerning the awareness of CPGs were excluded, leaving 3,343 for inclusion in the analysis. Among all included respondents, 611 (weighted% = 17.5%) answered that they knew of CPGs (Fig 1).

Table 1 shows the characteristics of respondents. Approximately one-fifth of respondents had made no healthcare visits in the preceding 12 months. Along with the degree of familiarity with CPGs, we divided respondents into three groups: 1) those who were not aware of CPGs (n = 2,732, weighted% = 82.5%), 2) those who were aware of CPGs but had never read one (n = 342, weighted% = 9.9%), and 3) those who were aware of CPGs and had read one (n = 259, weighted% = 7.6%). Supplementary S2 Table shows the associations between respondent characteristics and familiarity with CPGs. No associations were seen for age; marital status; employment status. Associations were observed between familiarity with the CPGs and other characteristics, namely for female, higher education, single-person household, annual household income of eight million yen or more, and history of any cancer. The absence of healthcare visits in the preceding year was related to lower familiarity.

**Table 1:** Characteristics of respondents.

	Total (n = 3,343)	
	n	weighted%
Age (years)		
20-29	263	11.4
30-39	388	12.3
40-49	571	17.7
50-59	599	17.0
60-69	635	14.6
≥ 70	877	27.1
Gender		
Male	1,504	48.5
Female	1,829	51.5
Education		
≤ High school graduate	1,525	46.3
Vocational school/2-year college	818	23.2
≥ University	976	30.5
Marital status		
Married	2,294	65.9
Not married	1,030	34.1
Number of household members		
1	478	15.5
≥ 2	2,829	84.5
Employment status		
Employed	2,078	63.1
Not employed	1,220	36.9
Annual household income (yen)		
< 4 million	1,250	37.9
4 million < 8 million	1,239	38.4
≥ 8 million	759	23.6
General health		
Excellent/very good	557	18.2
Good	1,944	57.5
Fair/poor	804	24.3
Healthcare visits per year		
None	676	21.4
1–4	1,056	32.9
5–9	675	20.6
≥ 10	852	25.1
Cancer history (self)		
Yes	355	10.3
No	2,978	89.7
Cancer history (family)		
Yes	1,967	58.4
No	1,336	41.6
Cancer history (friends)		
Yes	2,001	58.4
No	1,298	41.6
Diabetes (self)		
Yes	442	13.3
No	2,881	86.7
Hypertension (self)		
Yes	1,186	35.2
No	2,182	64.8
Heart disease (self)		
Yes	210	6.3
No	3,112	93.7
Cerebrovascular disease (self)		
Yes	51	1.5
No	3,256	98.5
Chronic lung disease (self)		
Yes	350	10.5
No	2,970	89.5
Arthritis (self)		
Yes	200	6.0
No	3,120	94.0
Depression (self)		
Yes	266	8.0
No	3,055	92.0

### Characteristics of respondents and awareness of clinical practice guidelines

Logistic regression analysis was used to examine factors associated with awareness of CPGs among respondents (Table 2). The model explained approximately 9.0% of the variance in awareness. Awareness was low in those with two or more household members (aOR = 0.601, 95% confidence interval (CI): 0.422–0.827, *P* = 0.004) and high in those with vocational school or university degrees (aOR = 2.060, 95% CI: 1.615–2.628, *P* < 0.001, and aOR = 1.813, 95% CI: 1.416–2.322, *P* < 0.001, respectively) or an annual household income of 8 million yen or more (aOR = 1.882, 95% CI: 1.397–2.536, *P* < 0.001).

**Table 2:** Characteristics of respondents and awareness of clinical practice guidelines (n = 3,343).

	aOR	95%CI	*P*
Age (years)			
20-29	Reference		
30-39	0.793	[0.516 - 1.220]	0.291
40-49	0.732	[0.488 - 1.098]	0.131
50-59	0.860	[0.570 - 1.298]	0.472
60-69	0.973	[0.640 - 1.479]	0.896
≥ 70	0.690	[0.426 - 1.118]	0.132
Gender			
Male	Reference		
Female	1.193	[0.950 - 1.498]	0.129
Education			
≤ High school graduate	Reference		
Vocational school/2-year college	2.060	[1.615 - 2.628]	<0.001
≥ University	1.813	[1.416 - 2.322]	<0.001
Marital status			
Not married	Reference		
Married	0.884	[0.670 - 1.165]	0.380
Number of household members			
1	Reference		
≥ 2	0.601	[0.427 - 0.845]	0.004
Employment status			
Not employed	Reference		
Employed	0.891	[0.687 - 1.156]	0.385
Annual household income (yen)			
< 4 million	Reference		
4 million < 8 million	1.225	[0.935 - 1.605]	0.140
≥ 8 million	1.882	[1.397 - 2.536]	<0.001
General health			
Excellent/very good	1.373	[1.059 - 1.781]	0.017
Good	Reference		
Fair/poor	1.098	[0.849 - 1.420]	0.474
Healthcare visits per year			
None	Reference		
1 to 4	1.549	[1.132 - 2.120]	0.006
5 to 9	1.477	[1.054 - 2.069]	0.023
≥ 10	1.741	[1.242 - 2.441]	0.001
Cancer history (self)			
No	Reference		
Yes	1.708	[1.243 - 2.347]	<0.001
Cancer history (family)			
No	Reference		
Yes	1.458	[1.180 - 1.802]	<0.001
Cancer history (friends)			
No	Reference		
Yes	1.514	[1.203 - 1.905]	<0.001
Diabetes (self)			
No	Reference		
Yes	1.055	[0.774 - 1.438]	0.734
Hypertension (self)			
No	Reference		
Yes	0.808	[0.635 - 1.029]	0.084
Heart disease (self)			
No	Reference		
Yes	1.049	[0.656 - 1.678]	0.842
Cerebrovascular disease (self)			
No	Reference		
Yes	0.854	[0.310 - 2.351]	0.759
Chronic lung disease (self)			
No	Reference		
Yes	1.208	[0.872 - 1.672]	0.255
Arthritis (self)			
No	Reference		
Yes	0.970	[0.609 - 1.547]	0.899
Depression (self)			
No	Reference		
Yes	1.000	[0.696 - 1.437]	0.998
Nagelkerke R Square	0.090		

Logistic regression analyses were conducted with awareness of clinical practice guidelines (CPGs) (Yes) as objective variable, and age, gender, education, marital status, number of household members, employee status, household income, general health, healthcare visits per year, cancer history (self), cancer history (family), cancer history (friends), diabetes (self), hypertension (self), heart disease (self), cerebrovascular disease (self), chronic lung disease (self), arthritis (self), and depression (self) as explanatory variables.

aOR: adjusted odds ratio, CI: confidence interval.

Respondents reporting excellent or very good general health were more likely to be aware of CPGs (aOR = 1.373, 95% CI: 1.059–1.781, *P* = 0.017). Respondents with 10 or more healthcare visits per year had the highest odds of awareness compared to those with no visits (aOR = 1.741, 95% CI: 1.242–2.441, *P* = 0.001). Respondents with a personal history of cancer were more likely to be aware of CPGs (aOR = 1.708, 95% CI: 1.243–2.347, *P* < 0.001). Odds ratios were also higher in those with a cancer history in family members (aOR = 1.458, 95% CI: 1.180–1.802, *P* < 0.001) and friends (aOR = 1.514, 95% CI: 1.203–1.905, *P* < 0.001). Experience of other diseases was not associated with increased awareness of CPGs.

### Characteristics of respondents and having read clinical practice guidelines

Table 3 shows the association between respondent characteristics and their experience of reading CPGs. Most variables that were associated with awareness of CPGs were not associated with reading CPGs. The only variable associated with reading was a self-history of cancer (aOR = 1.858, 95% CI: 1.120–3.083, *P* = 0.017).

**Table 3:** Characteristics of respondents and having read clinical practice guidelines (n = 601).

	aOR	95%CI	*P*
Age (years)			
20-29	Reference		
30-39	0.607	[0.277 - 1.330]	0.211
40-49	0.656	[0.300 - 1.438]	0.292
50-59	0.524	[0.239 - 1.150]	0.107
60-69	0.709	[0.310 - 1.626]	0.416
≥ 70	0.756	[0.317 - 1.801]	0.527
Gender			
Male	Reference		
Female	0.768	[0.516 - 1.144]	0.193
Education			
≤ High school graduate	Reference		
Vocational school/2-year college	1.153	[0.704 - 1.887]	0.571
≥ University	0.997	[0.617 - 1.611]	0.990
Marital status			
Not married	Reference		
Married	1.297	[0.760 - 2.212]	0.339
Number of household members			
1	Reference		
≥ 2	0.550	[0.293 - 1.032]	0.062
Employment status			
Not employed	Reference		
Employed	1.199	[0.761 - 1.891]	0.433
Annual household income (yen)			
< 4 million	Reference		
4 million < 8 million	1.406	[0.831 - 2.380]	0.203
≥ 8 million	1.604	[0.888 - 2.900]	0.117
General health			
Excellent/very good	1.614	[0.985 - 2.643]	0.057
Good	Reference		
Fair/poor	1.480	[0.936 - 2.342]	0.093
Healthcare visits per year			
None	Reference		
1–4	1.500	[0.854 - 2.636]	0.158
5–9	1.489	[0.790 - 2.808]	0.217
≥ 10	1.455	[0.788 - 2.685]	0.229
Cancer history (self)			
No	Reference		
Yes	1.858	[1.120 - 3.083]	0.017
Cancer history (family)			
No	Reference		
Yes	1.449	[0.977 - 2.148]	0.065
Cancer history (friends)			
No	Reference		
Yes	1.142	[0.751 - 1.735]	0.534
Nagelkerke R Square	0.068		

Logistic regression analyses were conducted with having read clinical practice guidelines (CPGs) (Yes) as objective variable, and age, gender, education, marital status, number of household members, employee status, household income, general health, healthcare visits per year, cancer history (self), cancer history (family) and cancer history (friends) as explanatory variables.

aOR: adjusted odds ratio, CI: confidence interval.

### Perceptions of clinical practice guidelines after reading them

Table 4 presents participant perceptions of CPGs after reading them, along with the association between reading CPGs and these perceptions. Among respondents who were aware of CPGs, only about 50% disagreed with Perceptions 1, 2, and 6.

**Table 4:** Reading and perceptions of clinical practice guidelines (n = 601).

	Total		Reading CPGs					
			Yes		No		aOR [95%CI] *	*P*
	n	weighted%	n	weighted%	n	weighted%		
Perception 1: They are written only for healthcare professionals.								
Disagree	295	51.1	157	59.9	138	44.0	1.744 [1.239 - 2.455]	0.002
Agree/DK	283	48.9	99	40.1	184	56.0		
Perception 2: “Weak recommendation” implies that there is no sufficient evidence.								
Disagree	265	46.5	128	50.7	137	43.0	1.361 [0.941 - 1.968]	0.101
Agree/DK	312	53.5	127	49.3	185	57.0		
Perception 3: Patients will no longer have to make their own decisions about examinations and treatments.								
Disagree	481	83.4	227	88.9	254	79.0	1.715 [1.002 - 2.936]	0.049
Agree/DK	99	16.6	30	11.1	69	21.0		
Perception 4: We cannot use examination or treatment that differs from the recommendation.								
Disagree	437	76.2	207	80.5	230	72.8	1.572 [1.036 - 2.384]	0.033
Agree/DK	139	23.8	49	19.5	90	27.2		
Perception 5: “Clinical practice guidelines” restrict access to advanced medical care.								
Disagree	440	76.6	214	84.2	226	70.5	1.816 [1.162 - 2.837]	0.009
Agree/DK	140	23.4	43	15.8	97	29.5		
Perception 6: One’s experiences or those of patients with the same illness are more reliable than “clinical practice guidelines”.								
Disagree	294	50.7	144	56.7	150	45.7	1.472 [0.999 - 2.169]	0.051
Agree/DK	285	49.3	113	43.3	172	54.3		
Perception 7: Consulting with healthcare providers about “clinical practice guidelines” makes the relationship worse.								
Disagree	442	76.8	216	84.7	226	70.4	2.301 [1.383 - 3.827]	0.001
Agree/DK	135	23.2	39	15.3	96	29.6		
Perception 8: They do not contain information that would help patients or their families.								
Disagree	398	70.0	198	78.5	200	63.1	1.954 [1.268 - 3.012]	0.003
Agree/DK	180	30.0	58	21.5	122	36.9		

aOR; adjusted odds ratio, CI; confidence interval, CPG; clinical practice guideline, DK; don’t know.

* Logistic regression analyses were conducted with each perception (Disagree) as objective variable, having read clinical practice guidelines (Yes) as explanatory variable, and age, gender, education, marital status, number of household members, employee status, household income, general health, healthcare visits per year, cancer history (self), cancer history (family) and cancer history (their friend) as adjusted variables in each model.

Among respondents who had read CPGs, more than half correctly disagreed with each of the perceptions. Disagreement among readers was highest for Perception 3 (88.9%), followed by Perceptions 7 (84.7%), 5 (84.2%), and 4 (80.5%). This is the same order as that of all people who were aware of CPGs. Of the eight perceptions, reading CPGs was associated with Perceptions 1, 3, 4, 5, 7, and 8. The strongest association was with Perception 7 (aOR = 2.301, 95% CI: 1.383–3.827, *P* = 0.001), followed by Perceptions 8 (aOR = 1.954, 95% CI: 1.268–3.012, *P* = 0.003), 5 (aOR = 1.816, 95% CI: 1.162–2.837, *P* = 0.009) and 1 (aOR = 1.744, 95% CI: 1.239–2.455, *P* = 0.002). Supplementary S3 Table shows detailed results of these logistic regression analyses.

## Discussion

This study of the awareness, reading, and perceptions of CPGs in a Japanese general population showed that awareness was associated with higher education, higher household income, excellent health, and frequent healthcare visits. A self-history of cancer was the only factor associated with both awareness and reading. Among those aware of CPGs, reading CPGs was associated with the rejection of ideas that CPGs restrict care or harm patient-provider relationships.

In this study, awareness of CPGs among the general population in Japan was 17.5%. Previous studies have reported considerably higher levels of awareness; however, those studies primarily surveyed patients, individuals currently receiving medical care, or healthcare professionals rather than the general public [18]. This distinction in study populations is important because CPG awareness tends to be higher among groups with stronger or more frequent engagement with healthcare services. For example, surveys reporting awareness levels above 50% were conducted among people undergoing medical treatment [34–36] or among individuals affiliated with organizations involved in developing CPGs [9,37]. Similarly, a study using questionnaires distributed through self-help groups for cancer patients and cancer-related institutions found that 45% of patients were aware of CPGs [17], and a Japanese survey of people who had visited a doctor within the past three months reported awareness of 26.5% [18]. These findings suggest that awareness declines as the population becomes less connected to healthcare settings, which is consistent with the relatively low awareness observed in our general population sample. Strategies to increase CPG awareness could include media releases involving the general public, distribution of patient-specific literature [38], and incorporation of CPGs in health literacy education curricula in schools [39].

In addition to these approaches, integrating basic CPG literacy into national health education campaigns may help raise population-level understanding of the purpose and value of CPGs. Nationwide health promotion initiatives, which increasingly emphasize health information access, prevention, and shared decision-making, provide a natural platform for introducing core concepts related to CPGs. Embedding CPG literacy within such programs could help reduce socioeconomic disparities in awareness, support more informed decision-making, and align with broader public health goals.

In this study, demographic and socioeconomic factors, such as education level, income, frequency of healthcare visits, and health perception, were associated with awareness of CPGs. Given findings that health literacy affects health directly or through the social determinants of health [40,41], our study highlights the importance of targeting individuals with lower education and income levels and fewer healthcare interactions, and those in less healthy states to improve the awareness of CPGs. For individuals with low levels of education or income, educational materials and interactive instructional approaches can be effective; however, prior research emphasizes that a thorough needs assessment is an essential first step [42]. Accordingly, efforts to increase awareness of clinical practice guidelines among these groups should begin with a systematic assessment of their needs. Interestingly, living in a household with two or more members was associated with lower CPG awareness. One possible explanation is that individuals in multi-person households may rely on family members for advice or support when health concerns arise, which may reduce the need to independently search for medical information, including CPGs. In contrast, people living alone may be more likely to seek health information themselves or communicate directly with healthcare professionals, increasing their chance of encountering CPGs. These mechanisms may help explain the negative association observed in our analysis.

Regarding disease experience, a history of cancer was significantly associated with awareness of CPGs. The statistically significant associations of other diseases with CPG awareness may not have been identified due to the small number of people experiencing the disease, particularly for conditions such as heart disease, cerebrovascular disease, arthritis, and depression. However, a more critical consideration is that cancer is a life-threatening disease that necessitates complex treatment decisions. Unlike many other chronic conditions, cancer treatment often involves multiple modalities, including surgery, chemotherapy, radiation therapy, and immunotherapy. Consequently, patients are highly motivated to seek reliable information to make informed decisions [43,44]. CPGs serve as a trusted source of evidence-based recommendations, facilitating shared decision-making between patients and their physicians [45]. This context may contribute to the higher awareness of CPGs among individuals with a history of cancer.

Although both respondents reporting excellent or very good health and those with a history of cancer showed higher awareness of CPGs, these findings likely reflect different mechanisms. People who perceive their health as excellent may be more proactive in maintaining their health and more inclined to independently seek information, thereby increasing their likelihood of encountering CPGs. In contrast, individuals with a history of cancer may have become familiar with CPGs through their interactions with healthcare professionals, participation in treatment decision-making, or exposure to patient education materials during their care. These variables represent distinct constructs and were adjusted for simultaneously in the regression analysis; thus, the observed associations reflect different pathways to greater CPG awareness rather than conflicting patterns.

A larger proportion of respondents who had read CPGs failed to correctly disagree with Perceptions 2 (“Weak recommendation” implies that there is no sufficient evidence) and 6 (One’s experiences or those of patients with the same illness are more reliable than “clinical practice guidelines”). This might mean that respondents had difficulty understanding the limitations of CPGs and their recommendations. Publicly accessible medical information websites should communicate not only the strengths but also the limitations of CPGs and their recommendations; healthcare professionals should be educated to explain them to their patients; and well-designed educational materials should be developed to enhance the public and patients’ knowledge of CPGs.

Although the present study was not designed to evaluate the effectiveness of specific types of educational material, prior research in health communication indicates that materials are more effective when they use plain language, incorporate visual aids, and are tailored to individuals’ health literacy levels [46–49]. Our findings suggest that such approaches may be particularly important for addressing misconceptions about the limitations of CPGs. Future studies will be needed to determine the most effective formats for CPG-related educational resources.

The current study has several limitations. The response rate was 35.3%, and respondents may have been biased toward people with a high level of awareness of medical information. Accordingly, awareness, reading, and perceptions of CPGs may be overestimated. The sample size of respondents who were aware of CPGs was small, so statistically significant associations between socioeconomic factors and reading and perceptions of CPGs might have been overlooked. Additionally, 109 respondents did not answer the question on CPG awareness, and this group may have included individuals with little or no familiarity with CPGs. Excluding these non-respondents may have led to a slight underestimation of the proportion of the population who were unaware of CPGs and could have introduced minor bias into the observed associations. The small Nagelkerke values for each model suggest a large proportion of the variance in awareness, reading, and perceptions remains unexplained and may be influenced by unmeasured factors. Our survey did not include several potentially important determinants of CPG awareness, such as health literacy, exposure to health-related media, and access to digital information sources. Future research incorporating these variables will be valuable for more comprehensively identifying the drivers of CPG awareness and perceptions. Because the broader INFORM Study 2023 includes a ‘Living with Cancer’ component, individuals with cancer experience may have been more likely to respond, and this contextual framing may have increased the salience of cancer-related issues. Although such differential participation could have modestly inflated the overall estimate of CPG awareness, the prevalence remained low, suggesting that limited awareness is a pervasive issue even under conditions potentially favorable to higher recognition. While this may have contributed to the strong association observed between cancer history and CPG awareness, it also represents a strength in that guideline awareness is particularly important for cancer, where treatment decision-making is uniquely complex. Furthermore, the survey was administered exclusively by postal mail, which may have led to the under-representation of younger or digitally active individuals who are less likely to respond to mailed questionnaires. Although we conducted a weighted analysis to account for non-responses to improve representativeness, the survey modality may have introduced selection bias, potentially inflating CPG awareness among individuals more responsive to traditional mail-based surveys. In addition, the study was conducted in a general population in Japan, and application to other regions should be done with caution.

## Conclusion

Awareness and reading experience of CPGs were significantly associated with demographic and socioeconomic factors. Tailored outreach and educational strategies targeting populations with low awareness and misconceptions could enhance the effective dissemination and implementation of CPGs, and ultimately empower patients and the public to make more informed healthcare decisions.

## Supporting information

S1 File Excerpt of survey items related to clinical practice guidelines. (DOCX)

S2 Table Characteristics of respondents in detail. (XLSX)

S3 Table Reading and perceptions of clinical practice guidelines. (XLSX)
