# Supplementary material for: Factors associated with awareness, reading and perceptions of clinical practice guidelines among the general population in Japan: A nationwide cross-sectional survey (INFORM Study 2023)
Source: PLoS One. 2026 Feb 12;21(2):e0343033. doi: 10.1371/journal.pone.0343033 (PMC12900357; doi:10.1371/journal.pone.0343033)
Supplement: S1 File — (DOCX) [file pone.0343033.s001.docx]

**S1 Supplementary Material: Excerpt of Survey Items Related to Clinical Practice Guidelines**

G: Clinical practice guidelines

**Q1.** "Clinical practice guidelines" are documents that provide recommendations for desirable examination and treatment methods based on evidence. Thus far, the Ministry of Health, Labour and Welfare and various academic societies have developed "clinical practice guidelines" for each disease to improve the quality of medical care. Are you familiar with "clinical practice guidelines"?

(Mark only one.)

| **1.** I’ve never heard of them (go to Q1 on page 11)  **2.** I know a little bit about it  **3.** I know what they are like |  |
| --- | --- |

(Q2-Q3 are asked to those who chose either "2" or "3" in Q1.)

**Q2.** Have you ever read "clinical practice guidelines" to learn about your disease or that of someone you know? (Mark only one.)

| **1.** I have read it | **2.** Never read it |  |
| --- | --- | --- |

**Q3.** Please answer the following question regarding your opinion on the "clinical practice guidelines."

(Mark only one for each.)

|  | Strongly  agree | Somewhat  agree | Not so much | Not at all | Don't know |  |
| --- | --- | --- | --- | --- | --- | --- |
| (1) They are written only for health care professionals | **1** | **2** | **3** | **4** | **5** |  |
| (2) "Weak recommendation" implies that there is no sufficient evidence. | **1** | **2** | **3** | **4** | **5** |  |
| (3) Patients will no longer have to make their own decisions about examinations and treatments. | **1** | **2** | **3** | **4** | **5** |  |
| (4) We cannot use examination or treatment that differs from the recommendation. | **1** | **2** | **3** | **4** | **5** | ⑫ |
| (5) "Clinical practice guidelines" restrict access to advanced medical care. | **1** | **2** | **3** | **4** | **5** | ⑬ |
| (6) One’s experiences or those of patients with the same illness are more reliable than "clinical practice guidelines." | **1** | **2** | **3** | **4** | **5** | ⑭ |
| (7) Consulting with health care providers about "clinical practice guidelines" makes the relationship worse. | **1** | **2** | **3** | **4** | **5** |  |
| (8) They do not contain information that would help patients or their families. | **1** | **2** | **3** | **4** | **5** | ⑯ |
